# Retraction: The role of LINC00284 in the development of thyroid cancer *via* its regulation of the microRNA-30d-5p-mediated ADAM12/Notch axis

**DOI:** 10.3389/fonc.2023.1209909

**Published:** 2023-04-26

**Authors:** Frontiers Editorial Office

Following publication, concerns were raised regarding the integrity of Figures 3L, 5A, 5B, 5C and 6A. The authors failed to provide a satisfactory explanation during the investigation, which was conducted in accordance with Frontiers’ policies. As a result, the data and conclusions of the article have been deemed unreliable and the article has been retracted.

This retraction was approved by the Chief Editors of Frontiers in Oncology and the Chief Executive Editor of Frontiers. The authors have not responded to correspondence regarding this retraction.

